# Supplementary material for: Colon adenocarcinoma-derived cells possessing stem cell function can be modulated using renin-angiotensin system inhibitors
Source: PLoS One. 2021 Aug 24;16(8):e0256280. doi: 10.1371/journal.pone.0256280 (PMC8384197; doi:10.1371/journal.pone.0256280)
Supplement: Attachment — Submitted filename: Response to the Reviwers and the Editors Comments.docx [file pone.0256280.s008.docx]

**Authors’ Response to Editor’s and Reviewer’s Comments**

Editor:

*This is an interesting study/perspective on renin-angiotensin system inhibitors in CRC stem cells.
The aim and topic are new, the experiments were well designed though the Figures are sometimes out of focus or not well stained.
On the other hans they are excessive so that several must be deleted (the badly stained in particular.
Moreover the length of the Introduction is also excessive and must be cut. It is much more long than the Discussion.
Therefore the Authors must put together some figures in panels and delete the bad ones, and put all figures in the correct order with respect to paragraphs.
Then reduce the Introduction to a third at least (remember that less is more).
Finally add missing data like those in figure 5.*

Reviewer #1:

*Authors present an interesting perspective on renin-angiotensin system inhibitors in CRC stem cells. The topic is quite new, experiments are well designed and conducted.*

*Nevertheless, too many figures and the excess of words make this manuscript difficult to read. Figures can be put together so that at each result paragraph correspond one figure only. this also with respect to figure order, which does not follow results order. Some data are missing in some figures, such as figure 5.*

**Authors’ response:** We thank you and the reviewer for your support and suggestions. We have taken on board the helpful comments and have revised our manuscript accordingly. We have reduced the word count including substantially shortening the Introduction section and reducing the number of figures (from 18 to 10) by creating multi-panel figures and presenting some of the data as additional supplementary figures. We trust that this is now acceptable.
